# What Is It?

**Published:** 1879-01

**Authors:** 


### WHAT IS IT?

The following is just at hand, from one who has been in the "Bisness" for three years, and yet feels that he wants to *know more;* of this there will be no difference of opinion among those who read the letter.

No one will even pretend that he is in any respect fit to enter a dental or medical college, and yet he can enter nine-tenths of the dental or medical schools in the country, without hinderance, so far as requirements are concerned; but to the letter:

S—— O——

Mr. J—— T—— Dec 2

An narbor Mic.

Dear Ser I wont your Catilog of Dentistry and I wont to no What Bookes I Wil haf too git to complet mi Business I Work at the Bisness Three years and I Wont to no what Bookes To get to complet me Ples Let me no and I Wil send for them hoping I may hear from you sune Yours Truley

